# Supplementary material for: International Consensus on Definition of Mild-to-Moderate Ulcerative Colitis Disease Activity in Adult Patients
Source: Medicina (Kaunas). 2023 Jan 16;59(1):183. doi: 10.3390/medicina59010183 (PMC9861955; doi:10.3390/medicina59010183)
Supplement: Supplementary file 1 [file medicina-59-00183-s001.zip › medicina-2151419-supplementary.pdf]

Supplementary Table S1. Statements excluded.

Proposed statements
The symptom-based items of the Clinical Activity Index (CAI) should be used to assess mild to moderate ulcerative colitis disease activity
The symptom-based items of the Disease Activity Index (DAI) should be used to assess mild to moderate ulcerative colitis disease activity
The presence of abdominal pain/cramps should be used to assess mild to moderate ulcerative colitis disease activity
The physician's global assessment (PGA) should be used to assess mild to moderate ulcerative colitis disease activity
The presence of temperature due to colitis should be used to assess mild to moderate ulcerative colitis disease activity
The presence of weight loss should be used to assess mild to moderate ulcerative colitis disease activity
The presence of nocturnal bowel movements should be used to assess mild to moderate ulcerative colitis disease activity
The endoscopic-based items of the Disease Activity Index (DAI) should be used to assess mild to moderate ulcerative colitis disease activity
The endoscopic-based items of the Endoscopic Index (EI) should be used to assess mild to moderate ulcerative colitis disease activity
The endoscopic-based items of the Ulcerative Colitis Disease Activity Index (UCDAI) should be used to assess mild to moderate ulcerative colitis disease activity
Disease location should be used to define mild to moderate disease activity
Histologic measurements are important to define mild to moderate ulcerative colitis
The Geboes Score is an appropriate instrument for classifying disease activity in mild to moderate ulcerative colitis
The Nancy Score is an appropriate instrument for classifying histologic disease activity in mild to moderate ulcerative colitis
The Robarts Histopathology Index is an appropriate instrument for classifying histologic disease activity in mild to moderate ulcerative colitis
The modified Riley Index is an appropriate instrument for classifying histologic disease activity in mild to moderate ulcerative colitis
The presence of neutrophils in the epithelium is sufficient to define mild to moderate ulcerative colitis
The presence of neutrophils in the epithelium is sufficient to define mild to moderate ulcerative colitis
The CRP level is an appropriate marker for classifying disease activity in mild to moderate ulcerative colitis
The following definitions of the Clinical Activity Index (CAI) should be used to define mild to moderate ulcerative colitis disease activity: CAI score >4 or CAI score ≥6 or CAI score <14 or CAI score of 6-12 or CAI score of 4-12
The following definitions of the Disease Activity Index (DAI) should be used to define mild to moderate ulcerative colitis disease activity: DAI score of 4-11 or DAI score of 3-9 including an endoscopic subscore ranging from 1 to 2 or DAI score of 3-11 or DAI score of 4-10 or DAI score of 3-10
The following definitions of the symptom-based items of the Ulcerative Colitis Disease Activity Index (UCDAI) should be used to define mild to moderate ulcerative colitis disease activity: UCDAI score of 4-10 including an endoscopic subscore of at least 1 and a PGA ≤2 or UCDAI score of 3-8 or UCDAI score of 6-8 or UCDAI score of 4-8
The American College of Gastroenterology Guidelines are appropriate for defining mild to moderate ulcerative colitis disease activity (<4 stools/day with intermittent blood in stools, mild occasional urgency, normal hemoglobin, ESR<30 mm/h, CRP elevated, FC >150-200, MES=1, UCEIS= 2-4)
The American Gastroenterology Association Guidelines are appropriate for defining mild to moderate ulcerative colitis disease activity (<4 stools/day with intermittent blood in stools, normal temperature,

normal pulse, normal hemoglobin, ESR≤30 mm/h, Non-colonic features on radiograph, none clinical signs)
The British Society of Gastroenterology Guidelines are appropriate for defining mild to moderate ulcerative colitis disease activity (Mayo score 3-5)
The European Crohn's and Colitis Organization Guidelines are appropriate for defining mild to moderate ulcerative colitis disease activity (<4 bloody stools/day, <90 bpm, <37.5°C temperature, >11.5 g/dL hemoglobin, ESR<20 mm/h, CRP normal)
Anxiety/depression measurements are important to define mild to moderate ulcerative colitis
